# *Cryptococcus neoformans *induces IL-8 secretion and CXCL1 expression by human bronchial epithelial cells

**DOI:** 10.1186/1465-9921-9-9

**Published:** 2008-01-22

**Authors:** Loïc Guillot, Scott F Carroll, Mohamed Badawy, Salman T Qureshi

**Affiliations:** 1 McGill Centre for the Study of Host Resistance, Room L11-403, 1650 Cedar Avenue, Montreal, QC, H3G 1A4, Canada; 2 Department of Human Genetics, McGill University, Montreal, QC, Canada; 3 Department of Medicine, McGill University, Montreal, QC, Canada; 4 Faculty of Medicine, Université de Montréal, Montreal, QC, Canada

## Abstract

**Background:**

*Cryptococcus neoformans *(*C. neoformans*) is a globally distributed fungal pathogen with the potential to cause serious disease, particularly among immune compromised hosts. Exposure to this organism is believed to occur by inhalation and may result in pneumonia and/or disseminated infection of the brain as well as other organs. Little is known about the role of airway epithelial cells in cryptococcal recognition or their ability to induce an inflammatory response.

**Methods:**

Immortalized BEAS-2B bronchial epithelial cells and primary normal human bronchial epithelium (NHBE) were stimulated *in vitro *with encapsulated or acapsular *C. neoformans *cultivated at room temperature or 37°C. Activation of bronchial epithelial cells was characterized by analysis of inflammatory cytokine and chemokine expression, transcription factor activation, fungal-host cell association, and host cell damage.

**Results:**

Viable *C. neoformans *is a strong activator of BEAS-2B cells, resulting in the production of the neutrophil chemokine Interleukin (IL)-8 in a time- and dose-dependent manner. IL-8 production was observed only in response to acapsular *C. neoformans *that was grown at 37°C. *C. neoformans *was also able to induce the expression of the chemokine CXCL1 and the transcription factor CAAT/enhancer-binding protein beta (CEBP/β) in BEAS-2B cells. NHBE was highly responsive to stimulation with *C. neoformans*; in addition to transcriptional up regulation of CXCL1, these primary cells exhibited the greatest IL-8 secretion and cell damage in response to stimulation with an acapsular strain of *C. neoformans*.

**Conclusion:**

This study demonstrates that human bronchial epithelial cells mediate an acute inflammatory response to *C. neoformans *and are susceptible to damage by this fungal pathogen. The presence of capsular polysaccharide and *in vitro *fungal culture conditions modulate the host inflammatory response to *C. neoformans*. Human bronchial epithelial cells are likely to contribute to the initial stages of pulmonary host defense *in vivo*.

## Background

*Cryptococcus neoformans *(*C. neoformans*) has been recognized as an important emerging fungal pathogen throughout the world during the past two decades [1]. Healthy individuals frequently develop asymptomatic or mild infection with *C. neoformans *while humans with impaired host defenses may progress to severe pneumonia and potentially fatal meningoencephalitis [2]. Natural infection is believed to occur via inhalation and is usually caused by an encapsulated cryptococcal strain, although recent investigations have reported similar clinical disease caused by acapsular yeast forms [3,4]. The use of animal models such as genetically engineered or naturally mutant mice have shown that the protective host immune response against *C. neoformans *requires type 1 helper T (Th1)-cell mediated immunity characterized by activation of CD4^+ ^and CD8^+ ^T cells and secretion of the Th1-related cytokines gamma interferon (IFN-γ), IL-12, IL-18, and tumor necrosis factor alpha (TNF-α) [5]. A variety of other cell populations including B cells [6], natural killer (NK), NKT, gamma-delta antigen receptor-bearing T (γδT) cells [7] and dendritic cells [8] have also been implicated in the host immune response against *C. neoformans*. Furthermore, several investigations have defined a major role for lung alveolar macrophages in the initial host response against *C. neoformans *[9,10]. While there is little doubt that the alveolar macrophage is a key mediator of host immunity against *C. neoformans *and other pulmonary pathogens [11], the role of pulmonary epithelial cells in resistance to cryptococcal infection has not been well-characterized.

The lung epithelium represents much more than a simple protective physical barrier between the external environment and underlying tissues. In fact, both constitutive and inducible defense mechanisms of the airway lining are now recognized as fundamental elements of an effective antimicrobial environment [12]. The role of the airway lining as a highly responsive and multifunctional interface in the host innate immune response against various microorganisms has been summarized in several recent reviews [12,13]. *C. neoformans *has been shown to bind to the A549 human alveolar cell line in a time- and temperature-dependent manner, with apparent internalization [14]. This report demonstrated that factors such as yeast culture age and *in vitro *growth conditions influenced lung epithelial cell binding by various strains of *C. neoformans *and showed greater adherence to A549 cells in the absence of a polysaccharide capsule [14]. Using the same cell line, another group recently reported that purified GXM, the major capsular component of *C. neoformans*, could induce IL-8 secretion after binding to the CD14 receptor [15,16]. Finally, the multifunctional enzyme secretory phospholipase B (PLB1) has also been shown to play a role in the adhesion process of *C. neoformans *to the alveolar epithelium [17]. To the best of our knowledge, there have been no studies characterizing the interaction between *C. neoformans *and cells of the airway lining that represent a first site of contact for airborne pathogens. Therefore, we investigated the ability of immortalized and normal human bronchial epithelial cells to trigger a host inflammatory response to viable *C. neoformans*. We found that BEAS-2B cells produced the potent chemokine IL-8 in an AP-1 and NF-κB dependent manner when stimulated with acapsular *C. neoformans*. The presence of acapsular *C. neoformans *also led to an increase in expression of the chemokine CXCL1 as well as the transcription factor CAAT/enhancer-binding protein beta (CEBP/β). Primary Normal Human Bronchial Epithelial cells also secreted more IL-8 and exhibited significantly greater cell damage in response to acapsular *C. neoformans *compared to stimulation with an encapsulated strain. Together these data clearly demonstrate that airway epithelial cells mount a strong inflammatory response to *C. neoformans *that is modulated by the presence of the polysaccharide capsule.

## Methods

### Reagents and antibodies

F-12K nutrient mixture (Kaighn's modification), penicillin/streptomycin, glutamine, and trypsin-EDTA were from GIBCO Life Technologies, Ltd (Paisley, UK). Fetal calf serum (FCS) was from Hyclone (Logan, UT). Human recombinant TNF-α was from R&D systems (Minneapolis, MN). Lipopolysaccharide (LPS) (*E. Coli*, O55:B5) and fluorescein isothiocyanate (FITC) were from Sigma (Oakville, ON). Diff-Quik^® ^stain set was from Dade Behring (Newark, DE).

### *C. neoformans *strains

Wild type B3501, mutant CAP64, and 52D cryptococcal strains (34873, 52816, and 24067, respectively) were obtained from the American Type Culture Collection (ATCC, Manassas, VA). CAP64 is an avirulent capsule-deficient mutant [18,19] derived from the parental laboratory strain B3501. Strain 52D is a moderately virulent clinical isolate from human cerebrospinal fluid. Capsular and acapsular forms of *C. neoformans *were grown and maintained on Sabouraud dextrose agar (BD, Sparks, MD). For cell stimulation, a single colony suspension in Sabouraud dextrose broth (BD, Sparks, MD) was prepared and grown to early stationary phase (48 h) at room temperature or 37°C with continuous rotation. The culture was then washed with PBS, counted on a hemacytometer, and diluted to the desired concentration in cell culture media.

### Cells and culture conditions

The BEAS-2B human tracheobronchial epithelial cell line was obtained from the ATCC (CRL-9609) and cultured as described previously [20]. For stimulation experiments, cells were seeded at 2 × 10^5 ^cells on 12-well plates (Costar, New York, NY) and grown at 37°C in 5% CO_2_. Forty-eight hours later, cells were washed once and triplicate wells were stimulated with various multiplicities of infection (MOI) as described in the figure legends. NHBE (Normal Human Bronchial Epithelial) cells from Cambrex (Walkersville, MD) were maintained at 37°C and 5% CO_2 _and subcultured in Bronchial Epithelial Growth Medium (BEGM) as recommended by the manufacturer. For stimulation, NHBE were seeded at 10000 cells/cm^2 ^in 12-well plate (Costar). Forty-eight hours later, cells were counted and triplicate wells were stimulated with an MOI of 20 of acapsular CAP64 or capsular 52D *C. neoformans*.

### RT-PCR

Total RNA was extracted using an RNeasy kit (Qiagen, Mississauga). Reverse transcription (RT) was performed with 0.5 μg of total RNA that had been extracted, using the ABI high capacity cDNA archive kit (ABI, Foster City, CA). PCR was performed using specific primers (AlphaDNA, Montreal, QC) for human CEBP/β (sense: 5'-GAC AAG CAC AGC GAC GAG TA-3'; antisense: 5'-AGC TGC TCC ACC TTC TTC TG-3' – amplicon size 158 bp), CXCL-1 (sense: 5'-AGG GAA TTC ACC CCA AGA AC-3'; antisense: 5'-CAC CAG TGA GCT TCC TCC TC-3' – amplicon size 204 bp), CCL2 (sense: 5'-TCC AGC ATG AAA GTC TCT GC-3'; antisense: 5'-TGG AAT CCT GAA CCC ACT TC-3'-amplicon size 265 bp); the CCL15 primer set was obtained from SuperArray – amplicon size 150 bp. As an internal control, we used primers for the detection of human β-actin (sense 5'-AAG GAG AAG CTG TGC TAC GTC GC-3'; antisense 5'-AGA CAG CAC TGT GTT GGC GTA CA-3' – amplicon size 266 bp [20]). PCR amplifications were performed in a Peltier thermal cycler apparatus (MJ Research, Watertown, MA) using the Amplitaq polymerase (ABI, Foster City, CA). For the detection of CEBP/β, CXCL1 and CCL2 the thermocycling protocol was: 95°C for 1 min, 30 cycles of denaturation at 95°C for 45 s, annealing at 56°C for 45 s, and extension at 72°C for 1 min. For the detection of CCL15, 34 cycles were applied; for the detection of β-actin, 24 cycles were applied. Amplification products were resolved on a 1.5% agarose gel containing ethidium bromide and recorded with a Gene Genius bioimaging system (Syngene, Frederick, MD). Different cycle numbers for each target were performed to verify that each PCR product was analyzed during the exponential phase of the amplification reaction.

### Real time PCR

Real-time PCR was performed using an ABI Prism 7500 Real time PCR System (Applied Biosystems, Foster City, CA). Each reaction contained 10 μl of 2× platinum^® ^SYBR^® ^Green PCR Supermix (including Platinum^® ^Taq polymerase, SYBR^® ^green dye, Tris-HCL, KCL, 6 mM MgCl_2_, 400 μM dATP, 400 μM dCTP, 400 μM dGTP, 800 μM dUTP, Uracil DNA glycosylase (UDG) and stabilizers) (Invitrogen, Carlsbad, CA), 0.04 μl of ROX reference dye, 0.2 μM of each of forward and reverse primers (same as described above), and 25 ng of cDNA as template in a final volume of 20 μl. Reactions were incubated at 50°C for 2 min followed by 95°C for 10 min. The amplification profile was 15 s denaturation at 95°C followed by 40 s annealing at 60°C for a total of 40 cycles. Then a dissociation curve was realized to analyze the specificity of the reaction and the amplification of the expected single products was confirmed on 1.5% agarose gels stained with ethidium bromide (data not shown). Data were analyzed with the comparative Ct method (ΔΔCt) outlined in the ABI user manual with the 7500 system SDS software (Applied Biosystems). For the relative quantification, the amount of the targets CCL2, CXCL1 and CEBP/β were normalized to β-actin (endogenous gene) relative to unstimulated cells used as the calibrator and calculated using 2^-ΔΔCtCt^.

### Analysis of *C. neoformans *binding

BEAS-2B cells were seeded at 2 × 10^5 ^on individual coverslips (Fisher scientific, Pittsburg, PA) in a 12-well plate and stimulated with *C. neoformans *as indicated in the figure legends. Twenty-four hour later, cells were washed twice with PBS and then stained with Diff-Quik^® ^products. Slides images were captured with a Retiga 1300 C digital camera (QImaging Corp., Burnaby, BC) attached to a Zeiss AxioSkop II (Carl Zeiss Canada Ltd., Toronto, ON) light microscope.

### Fluorescence Activated Cell Sorter (FACS) Analysis

BEAS-2B cells were seeded at 2 × 10^5 ^on 12-well plates and grown for 48 h. Fluorescently labeled *C. neoformans *was prepared by incubation with 0.5 mg/ml FITC for 10 minutes, followed by three washes with PBS, as previously described [21]. BEAS-2B cells were incubated with *C. neoformans *for 3 hours, then washed twice with PBS, and trypsinized. Cells were subsequently incubated in the presence or absence of the extracellular dye quencher Trypan blue (200 μg/ml), washed three times with PBS, and their fluorescence was analyzed using a FACScalibur (BD Biosciences, San Jose, CA).

### Epithelial cell transfection and reporter gene studies

BEAS-2B cells were seeded at 7 × 10^4 ^on 24 well plates (Costar) 24 h before transfection using FuGENE 6 transfection reagent (Roche Molecular Diagnostics, Indianapolis, IN) according to the manufacturer's instructions. Cells were transfected with 200 ng of -133-luc, NFκB mutated-luc and AP-1-mutated-luc IL-8 luciferase constructions described elsewhere [22]. After 48 h, cells were left untreated or stimulated for 6 h with a MOI of 20 of acapsular *C. neoformans *or 20 ng/ml of TNF-α. Cell lysates were obtained by treatment with passive lysis buffer and firefly luciferase activity was measured as described previously [20], using a Lmax (Molecular Devices, Sunnyvale, CA) apparatus. Results are expressed as relative luciferase units (RLU).

### Gene Expression Array Studies

Human cytokine and receptor microarrays profiling a total of 113 cytokines, chemokines, and the corresponding receptor genes involved in the inflammatory response (Oligo GEArray^® ^OHS-011, SuperArray, Bethesda, MD) were used to evaluate the gene expression profile of BEAS-2B cells stimulated with *C. neoformans*. Total cellular RNA (0.8 μg) was used as the template to produce biotin-labeled amplified cRNA; subsequent hybridization of the microarrays was performed with 3 μg of biotin labeled cRNA. Microarray analysis was performed as manufacturer's recommendations. An image of each array was taken and saved using a Gene Genius bioimaging system (Syngene) followed by analysis using the GEArray Expression analysis software. The relative amount of each target gene transcript was estimated by comparing its signal intensity with the signal derived from two housekeeping genes (β-actin, GAPDH).

### Cytokine and LDH measurements

The levels of human IL-8 and lactate dehydrogenase (LDH) in cell culture supernatants were determined using a DuoSet ELISA kit (R&D systems, Minneapolis, MN) and a LDH assay kit (CytoTox 96^® ^Non-radioactive cytotoxicity assay, Promega, Madison, WI), respectively.

### Statistical Analysis

Each point corresponds to the mean ± S.D. of the indicated number of experiments. The statistical significance of single comparisons was analyzed using the unpaired Student's t test with a threshold of P ≤ 0.05. For multiple comparisons, statistical significance was determined by a one-way ANOVA with post-test comparisons using the Tukey test with a threshold of P ≤ 0.05.

## Results

### Acapsular *C. neoformans *stimulates IL-8 production by BEAS-2B cells

**Figure 1:**
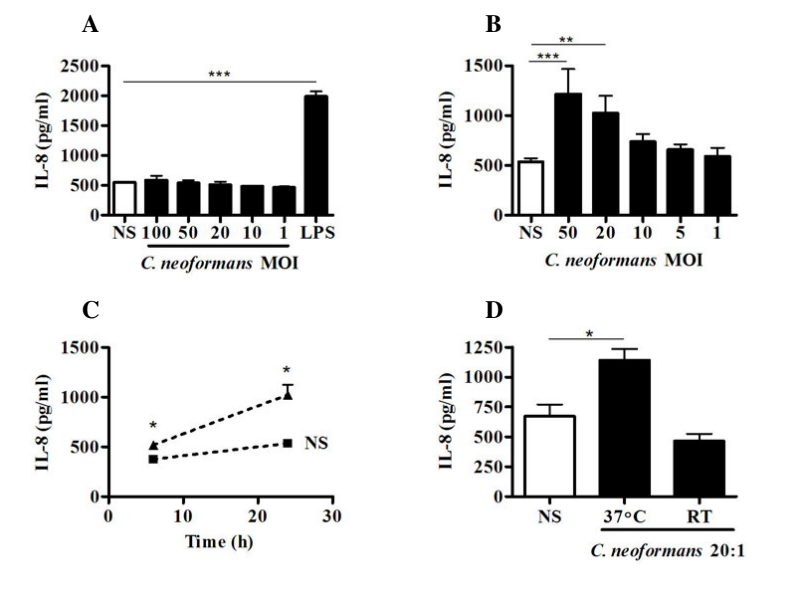
Acapsular *C. neoformans *induces IL-8 production by BEAS-2B cells in a dose, time, and temperature dependent manner. (A) BEAS-2B cells were unstimulated (NS, white box) or stimulated (black box) for 24 h with various MOI (100, 50, 20, 10, 1) of *C. neoformans *B3501 cultured at RT; LPS (1 μg/ml) was used as a positive stimulus. B) BEAS-2B cells were untstimulated (NS, white box) or stimulated for 24 h with various MOI (50, 20, 10, 5 and 1) of acapsular *C. neoformans*. C) BEAS-2B cells were stimulated with acapsular *C. neoformans *(MOI of 20) for 6 h and 24 h. (D) BEAS-2B cells were stimulated for 24 h with acapsular *C. neoformans *(MOI of 20) grown at 37°C or RT. The cell supernatants were collected and ELISA was used to measure IL-8 concentrations. All results are expressed as the mean ± S.D. of triplicate measurements and are representative of three independent experiments; *P ≤ 0.05, ** *P *≤ 0.01, *** *P *≤ 0.001 relative to NS.

To determine whether airway epithelial cells are able to mediate an anti-cryptococcal response, we measured IL-8 protein secretion by human BEAS-2B following *in vitro *stimulation with viable *C. neoformans*. In accordance with a previous study that demonstrated capsule- and temperature-dependent adhesion of *C. neoformans *to human alveolar epithelium [14], both encapsulated and unencapsulated yeast forms were grown at room temperature (RT) as well as 37°C prior to cell stimulation. All yeast cultures were grown to late log phase prior to use. The in vitro growth kinetic of *C. neoformans *was comparable between the encapsulated and unencapsulated forms; however, higher growth rates for both strains were achieved at room temperature (data not shown). Stimulation of BEAS-2B cells with the encapsulated B3501 strain did not induce significant release of IL-8, regardless of whether it was grown at RT (Figure 1A) or 37°C (data not shown). The same results were also observed following stimulation of BEAS-2B with the encapsulated 52D strain grown at RT and 37°C (data not shown). In contrast, acapsular *C. neoformans *cultured at 37°C triggered substantial secretion of IL-8 in a concentration- (Figure 1B) and time- (Figure 1C) dependent manner in BEAS-2B cells. The amount of IL-8 secretion was significantly increased with a MOI of 20 or greater (Figure 1B); this was observed by 6 h post-stimulation and accumulated in the culture medium for up to 24 h (Figure 1C). No IL-8 production was observed in response to acapsular *C. neoformans *that had been cultured at RT (Figure 1D).

### Acapsular *C. neoformans *binds and tightly associates with BEAS-2B cells

**Figure 2:**
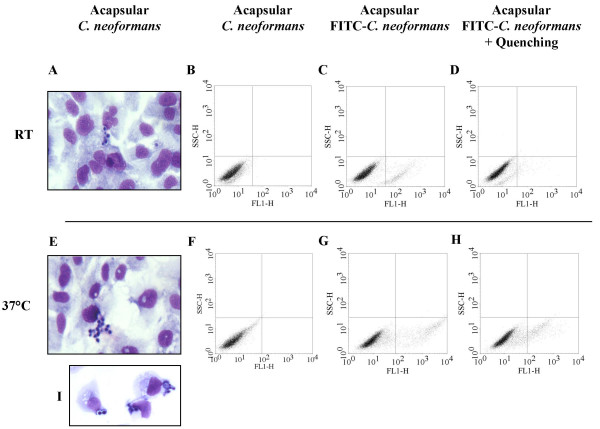
Acapsular *C. neoformans *binds and is internalized by BEAS-2B cells. Light microscopy of differentially stained BEAS-2B cells that were stimulated for 24 h with acapsular *C. neoformans *cultured at RT (A), 37°C (E), or 37°C followed by trypsinization (I). Flow cytometric analysis of BEAS-2B cells that were stimulated for 3 h with unlabeled (B and F) or FITC-labeled (C and G) acapsular *C. neoformans *grown at RT or 37°C respectively, and then quenched with trypan blue (D and H). Data are representative of three independent experiments.

Light microscopy of differentially stained co-cultures was used to characterize the interaction between *C. neoformans *and BEAS-2B cells. After washing and staining, a stable association of acapsular *C. neoformans *grown at RT or 37°C with BEAS-2B cells was observed (Figure 2). Interestingly, prominent *in vitro *aggregation of acapsular *C. neoformans *was observed when grown at 37°C (Figure 2E). The yeast-host cell interaction was resistant to trypsinization and appeared to be confined to the cell surface without evidence of internalization (Figure 2I). Conversely, no yeast-epithelial cell association was evident for the encapsulated form of *C. neoformans *(data not shown). A previously established technique to analyze the association and uptake of serotype A *C. neoformans *by human peripheral blood monocytes was then used to examine whether acapsular *C. neoformans *could be internalized by BEAS-2B cells [21]. We initially confirmed the efficacy of trypan blue to quench fluorescently labeled acapsular *C. neoformans*; FITC-labeled *C. neoformans *cells were homogenously fluorescent (99.5%) and this signal was almost completely eliminated (97.5%) by 200 μg/ml of trypan blue (data not shown). Incubation of BEAS-2B cells with FITC-labeled acapsular *C. neoformans *grown at RT or 37°C led to an increase in fluorescence (FL1-H) for 7.5% and 10.4% of BEAS-2B cells grown at RT or 37°C (Figure 2C and 2G, respectively). Following trypan blue treatment of BEAS-2B cells complexed with *C. neoformans *grown at RT, clear inhibition of the mean fluorescence intensity was observed (66.9% ± 4.5%; Figure 2D) and only 0.9% of cells retained the fluorescent label. This observation indicates that *C. neoformans *grown at RT is able to bind BEAS-2B cells but remains accessible to the quenching reagent. Comparable inhibition of the mean fluorescence intensity was observed following trypan blue treatment of BEAS-2B cells complexed with *C. neoformans *grown at 37°C (78.9 ± 0.7%; Figure 2H) however, 6.8% of BEAS-2B cells retained the fluorescent label, indicating that a small fraction of acapsular *C. neoformans *grown at 37°C is inaccessible to quenching by trypan blue.

### Acapsular *C. neoformans *induces mild LDH release from BEAS-2B cells

**Figure 3:**
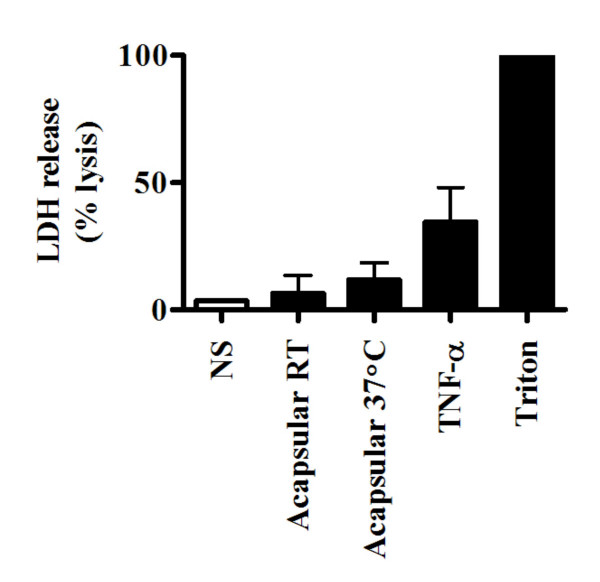
Acapsular *C. neoformans *induces mild LDH release by BEAS-2B cells. BEAS-2B cells were left untreated (NS, white box) or stimulated for 24 h with a MOI = 20 of acapsular *C. neoformans *grown at RT or 37°C or 50 ng/ml of TNF-α. Supernatants were collected and assayed for LDH release using colorimetry. Data represent the mean ± S.D. of triplicate measurements from two independent experiments and are expressed as a percentage of LDH release from unstimulated BEAS-2B cells treated with a detergent lysis solution (triton).

To determine whether *C. neoformans *is able to induce damage of bronchial epithelial cells, release of the intracellular enzyme LDH was measured following incubation of BEAS-2B cells with viable *C. neoformans *for 24 hours. As shown in Figure 3, acapsular *C. neoformans *grown at RT or 37°C induced a relatively small amount of LDH release by BEAS-2B cells (7 ± 7.1% and 12 ± 6.7%, respectively) that is indicative of mild cytotoxicity compared to cells that were completely lysed with Triton (100%). To confirm the validity of the assay using a relevant biological stimulus, BEAS-2B cells were also incubated with a high dose of TNFα (50 ng/ml), an inflammatory cytokine that is known to induce cell cytotoxicity [23]. As expected, TNFα induced substantial LDH release (35 ± 13.6%) by BEAS-2B cells.

### IL-8 activation in BEAS-2B cells largely involves the transcription factor AP-1 and NF-κB

**Figure 4:**
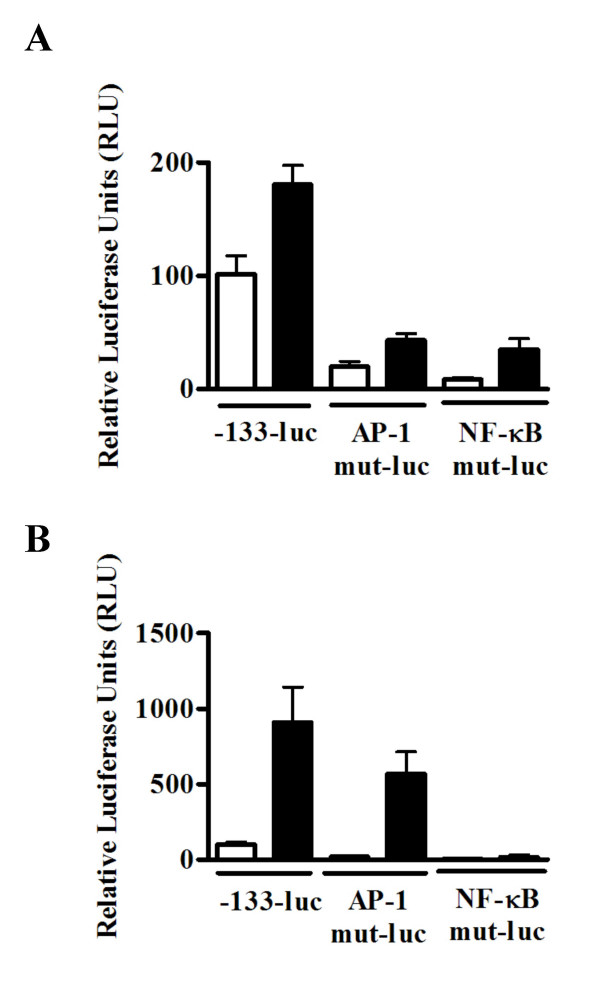
IL-8 activation by acapsular *C. neoformans *is dependent on NF-κB and AP-1. BEAS-2B cells were transiently transfected in duplicate with 200 ng of luciferase reporter plasmid DNA bearing the wild type IL-8 promoter (-133-luc), or mutations (mut) of its AP-1 (AP-1-mut-luc) or NF-κB (NF-κB-mut-luc) binding sites. After overnight incubation, cells were left untreated (white columns) or stimulated (black columns) for 6 h with acapsular *C. neoformans *(MOI = 20) (A) or TNF-α (20 ng/ml) (B). Cell lysates were collected and assayed for luciferase activity expressed as relative luciferase units (RLU). Data are shown as the mean ± S.D. Results are representative of three independent experiments.

Activation of transcription factors is required in many signal transduction pathways. For instance, IL-8 production can be activated in response to many different infectious or inflammatory conditions and is largely dependent on the transcription factor NF-κB and/or AP-1 [24]. To confirm whether these two signaling mechanisms are active in bronchial epithelial cells upon in vitro stimulation with *C. neoformans*, we examined IL-8 promoter activation using luciferase reporter plasmids bearing engineered mutations of either transcription factor-binding site. Consistent with the data obtained by ELISA, we detected IL-8 luciferase activity 6 h after stimulation with acapsular *C. neoformans *in BEAS-2B cells transfected with a wild-type IL-8 luciferase plasmid (-133-luc) (Figure 4A). A clear reduction of inducible IL-8 luciferase activity was observed following transfection of BEAS-2B cells with IL-8 reporter constructs mutated at the AP-1 (AP-1 mut-luc) or NF-κB (NF-κB mut-luc) binding sites (Figure 4A). Under these conditions we also observed an inhibition of basal IL-8 luciferase activity in unstimulated wells which is consistent with the observation that BEAS-2B cells constitutively release a detectable amount of IL-8 protein (Figure 1). To confirm the specificity of the mutated plasmid constructs, TNFα-induced stimulation of IL-8 reporter activity in BEAS-2B cells was performed as a positive control for IL-8 induction [24]. Consistent with previous reports using airway epithelial cells, we observed strong IL-8 luciferase activity in response to TNFα that was primarily dependent on NF-κB with a minor contribution of AP-1 [25] (Figure 4B).

### *C. neoformans *induces expression of the chemokine CXCL1 and the transcription factor CEBP/β in BEAS-2B cells

**Figure 5:**
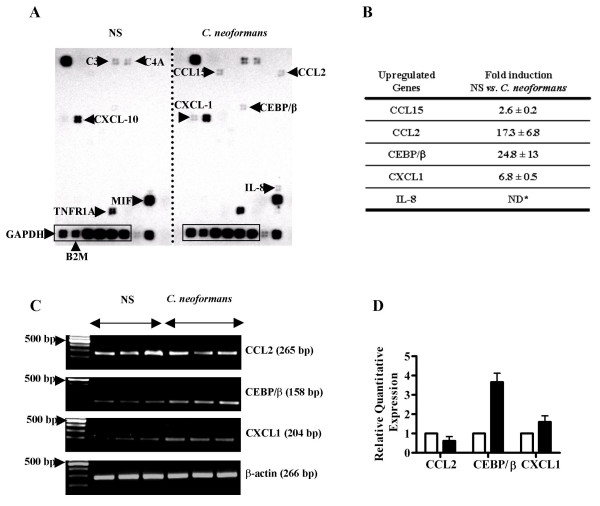
BEAS-2B cells induce chemokine gene expression in response to stimulation with acapsular *C. neoformans*. (A) BEAS-2B cells were left unstimulated (NS) (left panel) or stimulated (right panel) with acapsular *C. neoformans *MOI = 20 cultured at 37°C. Twenty-four hours later, RNA was extracted and analyzed by microarray for expression of inflammatory cytokines and their receptors. A box indicates the location of endogenous control (housekeeping) genes; glyceraldehyde-3-phosphate dehydrogenase (GAPDH) and beta-2 microglobulin (B2M) were used for normalization. (B) Summary of relative gene expression following stimulation with *C. neoformans*. Data shown is representative of three independent experiments. *Not determined due to undetectable IL-8 expression in untreated cells. (C) Expression of CCL2, CXCL1, and CEBP/β was analyzed by RT-PCR; β-actin was used as an endogenous control. (D) Relative quantification of CXCL1, CCL2, and CEBP/β expression in untreated (white columns) and stimulated (black columns) BEAS-2B cells was determined by real time PCR. Results are representative of three independent experiments.

**Figure 6:**
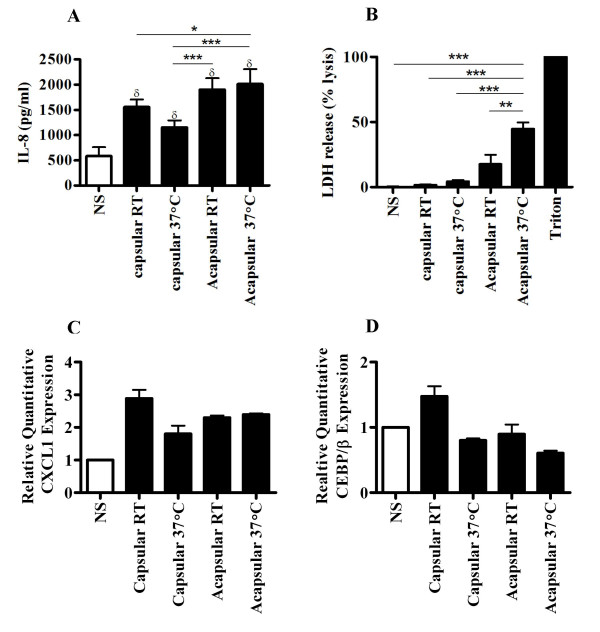
NHBE cells induce chemokine expression and are susceptible to damage following stimulation with *C. neoformans*. (A) NHBE cells were left unstimulated (NS, white box) or stimulated with a MOI = 20 of capsular or acapsular *C. neoformans *cultured at RT or 37°C, as indicated. Twenty-four hours later, supernatants were collected and ELISA was used to measure IL-8 concentrations. Results are expressed as the mean ± S.D. of triplicate measurements and are representative of three independent experiments. (B) Supernatants were also assayed for LDH release using colorimetry. Data are expressed as a percentage of LDH release from unstimulated cells treated with a detergent lysis solution (triton) and represent the mean ± S.D. of triplicate measurements from two independent experiments. (C, D) Relative quantification of CXCL1 and CEBP/β expression was determined by real time PCR. Results are representative of the mean ± S.D. of one experiment performed in triplicate; δ *P *≤ 0.05 vs. NS, * *P *≤ 0.05, ** *P *≤ 0.01, *** *P *≤ 0.001.

To further characterize the activation profile of bronchial epithelial cells by C. neoformans, we used an oligonucleotide microarray in order to examine the induction of a panel of 113 inflammatory cytokines, chemokines, and their receptors (a complete list of genes represented on the microarray is available on request). In unstimulated cells, we observed constitutive expression of C3, C4A, CXCL-10, MIF and TNFR1A that was not significantly influenced by C. neoformans stimulation (Figure 5A). Twenty-four hours after stimulation with acapsular *C. neoformans*, we observed the induction of IL-8, as expected, as well as up-regulation of CCL2, CXCL1, CCL15, and CEBP/β (Figure 5A and 5B). To validate these observations, we analyzed the expression of CXCL1, CCL2, and CEBP/β, and CCL15 by qualitative and real-time PCR. As shown in Figures 6C and 6D, we confirmed modest up-regulation of CXCL-1 and CEBP/β, but not CCL2, in *C. neoformans *stimulated cells compared to untreated BEAS-2B cells. Surprisingly, we were not able to demonstrate the expression of CCL15 either in unstimulated or *C. neoformans*-stimulated BEAS-2B cells by RT-PCR; nevertheless, we were able to weakly amplify CCL15 using a human reference cDNA as a positive control (kindly provided by SuperArray; data not shown). The integrity of all RNA preparations was verified by RT-PCR analysis of β-actin expression.

### *C. neoformans *is able to activate and damage primary NHBE cells

To exclude the possibility that IL-8 secretion and LDH release by BEAS-2B cell line in response to *C. neoformans *was a consequence of the viral immortalization process, we studied the response of primary NHBE cells to stimulation with an encapsulated or acapsular strain of *C. neoformans*. To more closely model conditions of authentic infection, a moderately virulent encapsulated clinical isolate, strain 52D, was used to stimulate primary cells. The NHBE cells used in this study were derived from a single male donor and had been extensively tested to exclude the presence of infectious agents. Interestingly, NHBE cells were highly responsive to *C. neoformans *and produced a significant amount of IL-8 in response to all conditions tested in comparison to NS (Figure 6A). Consistent with the observations using BEAS-2B cells, the maximal IL-8 secretion by NHBE cells was elicited by acapsular *C. neoformans*. In contrast to the BEAS-2B cell line, primary NHBE were activated by both acapsular and capsular *C. neoformans*, regardless of whether they were grown at RT or at 37°C. Nevertheless, we observed that the capsular *C. neoformans *grown at 37°C induced significantly lower IL-8 secretion in comparison to the acapsular strain grown at RT or 37°C. In contrast, capsular *C. neoformans *grown at RT elicited significantly lower IL-8 secretion only in comparison to acapsular *C. neoformans *grown at 37°C. NHBE cells were also highly susceptible to damage following a 24-hour incubation with viable *C. neoformans*. As shown in figure 6B, acapsular *C. neoformans *grown at 37°C induced the most significant LDH release by NHBE cells (44.6 ± 5%) in comparison to all other conditions, including the level elicited by acapsular *C. neoformans *grown at RT (17.6 ± 7%). Notably, capsular *C. neoformans *grown at RT or 37°C induced a relatively low amount of LDH release from NHBE cells (1.6 ± 0.65% and 4.3 ± 1.2, respectively). Finally, we examined transcriptional up regulation of CXCL1 and CEBP/β by *C. neoformans *in NHBE cells. Using real-time PCR, we observed a 2- to 3-fold induction of CXCL1 gene expression in NHBE by both capsular and acapsular *C. neoformans *grown at RT or 37°C (Figure 6C); however, no clear induction of CEBP/β was demonstrable under any of the experimental conditions used (Figure 6D).

## Discussion

It is clearly established that humans acquire *C. neoformans *infection from the environment, most likely through the inhalation of dehydrated or poorly encapsulated yeast particles termed infectious propagules. A high serologic prevalence in certain geographic regions despite low clinical infection rates suggests that in many cases the initial infection is mild or completely asymptomatic [26]. In the human lung, the host response to *C. neoformans *is presumed to start with alveolar macrophage activation, followed by the release of cytokines and chemokines that recruit inflammatory cells to the site of infection. In order for this interaction to occur, *C. neoformans *must transit the airways prior to reaching the alveolus, yet the responsiveness and contribution of the airway epithelium to host defense against cryptococcal infection is not well understood. The cells that line these passages have been increasingly recognized as an essential component of the host immune response [12,27]. The current study has shown that the interaction of *C. neoformans *with bronchial epithelial cells activates the expression of both transcription factors and chemokines that have well-established roles in host defense.

As an initial step in the investigation of human lung epithelial responses to viable *C. neoformans*, we selected the SV-40 immortalized BEAS-2B bronchial epithelial cell line that is derived from normal human tissue and capable of microbial recognition [13,28,29]. For a primary measure of cell activation, we quantified the secretion of IL-8, a prototypical neutrophil chemokine. Interestingly, we observed significant IL-8 secretion only when the BEAS-2B cell line was stimulated with an acapsular form of *C. neoformans*. Acapsular cryptococci exhibited a stable association with BEAS-2B cells that was resistant to enzymatic treatment with trypsin as well as various physical manipulations including washing and centrifugation of cell cultures. The well known anti-phagocytic and immunomodulatory properties of the polysaccharide capsule [3] are a plausible explanation for the absence of significant IL-8 release by BEAS-2B cells following stimulation with encapsulated *C. neoformans*, as well as the lack of visible fungal-host cell association under these conditions. In addition to the acapsular state, we observed that *C. neoformans *must be grown *in vitro *at 37°C in order to elicit IL-8 secretion by BEAS-2B cells. Environmental cues including temperature are well-known regulators of *C. neoformans *signal transduction that in turn influence microbial growth as well as virulence in animal models [30,31]. These data suggest that one or more temperature-regulated microbial factors distinct from GXM are required for activation of BEAS-2B cells. Alternatively, the *in vitro *cell aggregation that was observed at 37°C may also have contributed to the activation of BEAS-2B cells, possibly through coalescence or cross-linking of cryptococcal host cell surface receptors. This *in vitro *phenomenon has also been described for various other strains of *C. neoformans *[32-35] and has been associated with increased adherence as well as phagocytosis by mouse macrophages [34]. On the basis of these initial observations, we chose to focus subsequent investigations on the interaction of BEAS-2B cells with acapsular *C. neoformans*.

Internalization of *C. neoformans *by the A549 alveolar cell line has been demonstrated by two previous studies [14,15]. In one case, host cell damage following internalization of an encapsulated serotype A clinical isolate was also documented [15]. To determine whether BEAS-2B bronchial epithelial cells are also capable of fungal internalization, we first examined differentially stained co-cultures of *C. neoformans *and BEAS-2B cells by light microscopy. Despite the stable fungal-host cell interaction described above, no clear evidence of fungal internalization was directly visible. We then used a more sensitive flow cytometry technique combined with quenching of trypan blue dye to examine the interaction of BEAS-2B cells with FITC-labeled acapsular *C. neoformans *[21]. A clear increase in mean fluorescence intensity of BEAS-2B cells was observed following incubation with labeled cryptococci that were grown at 20° or at 37°C, verifying stable fungal binding in both conditions. The observation that only a minority of cells exhibited an increase in fluorescent signal may be attributable to partial disruption of fungal-host cell interactions by the washing, trypsinization, and centrifugation steps used to prepare the sample for cytometry. Quenching of fluorescence was almost complete for cryptococci grown at 20°C, a finding that is indicative of an extracellular fungal location. Interestingly, the residual fluorescence observed with fungi grown at 37°C indicates that they were either very tightly associated with the cell surface or may have been internalized by BEAS-2B cells. The extent of this process appeared to be quite limited as only a small percentage of fungi retained the trypan blue label. Further studies using a more detailed direct visualization technique such as electron microscopy will be required to confirm and discriminate between these two possibilities. Finally, limited damage of BEAS-2B cells by acapsular *C. neoformans *grown at 20°C or 37°C was observed by measurement of the intracellular enzyme LDH in cell supernatants following co-incubation.

In order to identify the signaling pathways that mediate IL-8 release by BEAS-2B cells in response to cryptococci, we performed transient transfections of a luciferase reporter plasmid downstream of the wild type human IL-8 promoter or engineered constructs bearing mutations of the NF-κB or AP-1 transcription factor binding sites. Compared to the wild type promoter sequence, luciferase activity was reduced for each of the mutant plasmids, demonstrating a role for both of these transcription factors in the up-regulation of IL-8 by *C. neoformans*. These observations are consistent with known regulation of IL-8 by other microbial pathogens [24]. We also detected up-regulation of several other inflammatory genes by *C. neoformans *in human BEAS-2B cells using an oligonucleotide microarray and confirmed the hybridization data by conventional and real-time PCR for CXCL1 and CEBP/β. CXCL1 has already been shown to be involved in leukocyte recruitment in mouse models of *C. neoformans *infection [36], indicating that activation of this chemokine is likely to be conserved between mice and humans. CEBP/β is expressed by alveolar and bronchial epithelium and is particularly involved in acute lung injury [37]. Interestingly, CEBP/β is also known to bind the IL-8 promoter in lung epithelial cells and activate transcription in a cooperative manner with NFκB [38]. Therefore, in addition to NF-κB and AP-1, CEBP/β activation in BEAS-2B cells upon stimulation with *C. neoformans *may also contribute to the up-regulation of IL-8 expression.

In order to confirm the authenticity of the observations that were obtained using BEAS-2B cells, we studied the effect of viable *C. neoformans *on primary NHBE cells that had not been subject to viral immortalization or serial *in vitro *passage. We found that primary NHBE cells were highly responsive to stimulation with *C. neoformans*. Specifically, for all experimental conditions tested (capsular or acapsular strains grown either at RT or 37°C), *C. neoformans *was able to stimulate the secretion of IL-8 and the expression of CXCL1 by NHBE. The highest level of IL-8 secretion was observed in response to acapsular *C. neoformans *grown *in vitro *at 37°C. At the mRNA level, CXCL1 expression did not appear to be influenced by *in vitro *growth conditions; however, this result does not exclude the possibility that both IL-8 and CXCL1 protein expression are coordinately regulated in response to *C. neoformans *stimulation. The confirmation of IL-8 protein secretion and CXCL1 up-regulation in NHBE strongly suggests that human bronchial epithelium recognizes *C. neoformans *and is capable of activating an *in vivo *host inflammatory response. Despite the observation that NHBE appeared to be responsive to capsular *C. neoformans*, growth of this organism at 37°C significantly diminished the magnitude of IL-8 secretion, suggesting that primary human epithelial cells respond to temperature dependent changes in fungal metabolism. Primary NHBE were also most susceptible to the cytotoxic effect of *C. neoformans*, especially when stimulated with the acapsular form that had been cultured at 37°C. Encapsulated *C. neoformans *elicited very little LDH from NHBE compared to acapsular form, regardless of whether it was cultivated at room temperature or 37°C. The fact that both chemokine activation and cell damage were reduced in the presence of the cryptococcal polysaccharide capsule points to an immunomodulatory role for GXM at the airway lining. It is tempting to speculate that following cryptococcal inhalation, induction of the GXM polysaccharide capsule expression in response to a change from ambient environmental conditions to human body temperature could facilitate the initial stage of fungal growth in the airways by modifying or diminishing the inflammatory response of the bronchial epithelium. Although the precise mechanism by which capsule alters the host response is not known, one possibility is that the presence of GXM polysaccharide modulates the interaction of cryptococcal surface or secreted structures with the bronchial epithelium. This relative suppression of host immunity mediated by *C. neoformans *might, in some cases, favor the development of progressive pulmonary infection. Finally, the observation that IL-8 secretion and cell damage were greatest for NHBE at 37°C also suggests that *C. neoformans *expresses unidentified, temperature-regulated structures potentially involved in pathogenesis.

Our data clearly demonstrate that both the immortalized BEAS-2B cell line and primary NHBE cells are activated by *C. neoformans*. Using either cell source, acapsular *C. neoformans *grown at 37°C elicited the maximal secretion of IL-8 and induced the greatest degree of cell damage. Transcriptional up regulation of CXCL1 was also exhibited by primary and immortalized cells in response to *C. neoformans*. The NHBE cells used in our report were somewhat more permissive to IL-8 secretion by encapsulated *C. neoformans *compared to BEAS-2B cells that were only activated by acapsular *C. neoformans *grown at 37°C. One other notable difference between primary and immortalized bronchial cells was the lack of CEBP/β up regulation by *C. neoformans *in NHBE. The precise reasons for these variations are not known but may be attributable either to the immortalization and maintenance of the BEAS-2B cell line or the inherent variability among human donors. Since the current data is derived from the study of cells from a single individual, it will be important to confirm these observations using a larger number of primary human samples.

In the lung, the inflammatory response of epithelial cells is likely integrated with alveolar macrophages to rapidly generate an innate immune response against *C. neoformans*. In this study, the amplitude of the IL-8 response was substantial, though relatively limited in comparison to highly pathogenic bacteria or viruses such as *Streptococcus pneumoniae *[39] or influenza virus [40], respectively. This modest inflammatory activation of the airway epithelium is consistent with the subtle clinical manifestations that are characteristic of cryptococcal respiratory tract infection in humans and may be one mechanism by which cryptococci are able to establish a latent infective state [2]. In contrast to studies with the A549 alveolar cell line [15,16], our results indicate that cryptococcal GXM polysaccharide down-regulates the activation of bronchial epithelial cells. We speculate that the acute inflammatory response may be even more limited among immune compromised hosts, resulting in insufficient airway cellular recruitment and a predisposition to the development of disseminated disease. Interestingly, modest IL-8 release was also recently demonstrated following the interaction of A549 cells with mycelial fragments and spores of *Aspergillus fumigatus *[41]. Taken together, these observations suggest that *C. neoformans *and perhaps other pathogenic fungi may escape the pulmonary innate immune response due to the limited extent of airway epithelial cell activation.

## Conclusion

Primary as well as immortalized human bronchial epithelial cells secrete IL-8 and up regulate the expression of CXCL1 following *C. neoformans *exposure. In addition, bronchial epithelial cells are susceptible to damage when stimulated with cryptococci. Activation and host airway cell damage appears to be modulated by capsule expression and the growth temperature of the infecting yeast. These host responses are very likely to represent an important component of the pulmonary innate immunity against this opportunistic pathogen.

## Competing interests

The author(s) declare that they have no competing interests.

## Authors' contributions

LG participated in the design of the study, performed cell culture and flow cytometry experiments, and drafted the manuscript. SC performed cell culture experiments, conducted statistical analysis, and drafted the manuscript. MB performed kinetic fungal culture experiments. SQ conceived of the study, supervised experiments, and critically revised the manuscript for important intellectual content. All contributors approved the final manuscript.

## References

[B1] Lin X, Heitman J (2006). The Biology of the Cryptococcus neoformans Species Complex. Annu Rev Microbiol.

[B2] Perfect JR, Casadevall A (2002). Cryptococcosis. Infect Dis Clin North Am.

[B3] Del Poeta M (2004). Role of phagocytosis in the virulence of Cryptococcus neoformans. Eukaryot Cell.

[B4] Torres HA, Prieto VG, Kontoyiannis DP, Raad (2005). Proven pulmonary cryptococcosis due to capsule-deficient Cryptococcus neoformans does not differ clinically from proven pulmonary cryptococcosis due to capsule-intact Cr. neoformans. Mycoses.

[B5] Huffnagle GB, Deepe GS (2003). Innate and adaptive determinants of host susceptibility to medically important fungi. Curr Opin Microbiol.

[B6] Rivera J, Zaragoza O, Casadevall A (2005). Antibody-mediated protection against Cryptococcus neoformans pulmonary infection is dependent on B cells. Infect Immun.

[B7] Kawakami K (2004). Regulation by innate immune T lymphocytes in the host defense against pulmonary infection with Cryptococcus neoformans. Jpn J Infect Dis.

[B8] Wozniak KL, Vyas JM, Levitz SM (2006). In vivo role of dendritic cells in a murine model of pulmonary cryptococcosis. Infect Immun.

[B9] Levitz SM (1994). Macrophage-Cryptococcus interactions. Immunol Ser.

[B10] Shao X, Mednick A, Alvarez M, van Rooijen N, Casadevall A, Goldman DL (2005). An innate immune system cell is a major determinant of species-related susceptibility differences to fungal pneumonia. J Immunol.

[B11] Zhang P, Summer WR, Bagby GJ, Nelson S (2000). Innate immunity and pulmonary host defense. Immunol Rev.

[B12] Diamond G, Legarda D, Ryan LK (2000). The innate immune response of the respiratory epithelium. Immunol Rev.

[B13] Hippenstiel S, Opitz B, Schmeck B, Suttorp N (2006). Lung epithelium as a sentinel and effector system in pneumonia--molecular mechanisms of pathogen recognition and signal transduction. Respir Res.

[B14] Merkel GJ, Scofield BA (1997). The in vitro interaction of Cryptococcus neoformans with human lung epithelial cells. FEMS Immunol Med Microbiol.

[B15] Barbosa FM, Fonseca FL, Holandino C, Alviano CS, Nimrichter L, Rodrigues ML (2006). Glucuronoxylomannan-mediated interaction of Cryptococcus neoformans with human alveolar cells results in fungal internalization and host cell damage. Microbes Infect.

[B16] Barbosa FM, Fonseca FL, Figueiredo RT, Bozza MT, Casadevall A, Nimrichter L, Rodrigues ML (2007). Binding of glucuronoxylomannan to the CD14 receptor in human A549 alveolar cells induces interleukin-8 production. Clin Vaccine Immunol.

[B17] Ganendren R, Carter E, Sorrell T, Widmer F, Wright L (2006). Phospholipase B activity enhances adhesion of Cryptococcus neoformans to a human lung epithelial cell line. Microbes Infect.

[B18] Chang YC, Penoyer LA, Kwon-Chung KJ (1996). The second capsule gene of cryptococcus neoformans, CAP64, is essential for virulence. Infect Immun.

[B19] Still CN, Jacobson ES (1983). Recombinational mapping of capsule mutations in Cryptococcus neoformans. J Bacteriol.

[B20] Guillot L, Medjane S, Le-Barillec K, Balloy V, Danel C, Chignard M, Si-Tahar M (2004). Response of human pulmonary epithelial cells to lipopolysaccharide involves Toll-like receptor 4 (TLR4)-dependent signaling pathways: evidence for an intracellular compartmentalization of TLR4. J Biol Chem.

[B21] Chaka W, Scharringa J, Verheul AF, Verhoef J, Van Strijp AG, Hoepelman IM (1995). Quantitative analysis of phagocytosis and killing of Cryptococcus neoformans by human peripheral blood mononuclear cells by flow cytometry. Clin Diagn Lab Immunol.

[B22] Ishikawa Y, Mukaida N, Kuno K, Rice N, Okamoto S, Matsushima K (1995). Establishment of lipopolysaccharide-dependent nuclear factor kappa B activation in a cell-free system. J Biol Chem.

[B23] Agarwal S, Drysdale BE, Shin HS (1988). Tumor necrosis factor-mediated cytotoxicity involves ADP-ribosylation. J Immunol.

[B24] Hoffmann E, Dittrich-Breiholz O, Holtmann H, Kracht M (2002). Multiple control of interleukin-8 gene expression. J Leukoc Biol.

[B25] Lakshminarayanan V, Drab-Weiss EA, Roebuck KA (1998). H2O2 and tumor necrosis factor-alpha induce differential binding of the redox-responsive transcription factors AP-1 and NF-kappaB to the interleukin-8 promoter in endothelial and epithelial cells. J Biol Chem.

[B26] Goldman DL, Khine H, Abadi J, Lindenberg DJ, Pirofski L, Niang R, Casadevall A (2001). Serologic evidence for Cryptococcus neoformans infection in early childhood. Pediatrics.

[B27] Bals R, Hiemstra PS (2004). Innate immunity in the lung: how epithelial cells fight against respiratory pathogens. Eur Respir J.

[B28] Sha Q, Truong-Tran AQ, Plitt JR, Beck LA, Schleimer RP (2004). Activation of airway epithelial cells by toll-like receptor agonists. Am J Respir Cell Mol Biol.

[B29] Amstad P, Reddel RR, Pfeifer A, Malan-Shibley L, Mark GE, Harris CC (1988). Neoplastic transformation of a human bronchial epithelial cell line by a recombinant retrovirus encoding viral Harvey ras. Mol Carcinog.

[B30] Mitchell TG, Perfect JR (1995). Cryptococcosis in the era of AIDS--100 years after the discovery of Cryptococcus neoformans. Clin Microbiol Rev.

[B31] Odom A, Muir S, Lim E, Toffaletti DL, Perfect J, Heitman J (1997). Calcineurin is required for virulence of Cryptococcus neoformans. Embo J.

[B32] Fries BC, Goldman DL, Cherniak R, Ju R, Casadevall A (1999). Phenotypic switching in Cryptococcus neoformans results in changes in cellular morphology and glucuronoxylomannan structure. Infect Immun.

[B33] Goldman DL, Fries BC, Franzot SP, Montella L, Casadevall A (1998). Phenotypic switching in the human pathogenic fungus Cryptococcus neoformans is associated with changes in virulence and pulmonary inflammatory response in rodents. Proc Natl Acad Sci U S A.

[B34] Li L, Zaragoza O, Casadevall A, Fries BC (2006). Characterization of a flocculation-like phenotype in Cryptococcus neoformans and its effects on pathogenesis. Cell Microbiol.

[B35] Wormley FL, Heinrich G, Miller JL, Perfect JR, Cox GM (2005). Identification and characterization of an SKN7 homologue in Cryptococcus neoformans. Infect Immun.

[B36] Kawakami K, Shibuya K, Qureshi MH, Zhang T, Koguchi Y, Tohyama M, Xie Q, Naoe S, Saito A (1999). Chemokine responses and accumulation of inflammatory cells in the lungs of mice infected with highly virulent Cryptococcus neoformans: effects of interleukin-12. FEMS Immunol Med Microbiol.

[B37] Cassel TN, Nord M (2003). C/EBP transcription factors in the lung epithelium. Am J Physiol Lung Cell Mol Physiol.

[B38] Matsusaka T, Fujikawa K, Nishio Y, Mukaida N, Matsushima K, Kishimoto T, Akira S (1993). Transcription factors NF-IL6 and NF-kappa B synergistically activate transcription of the inflammatory cytokines, interleukin 6 and interleukin 8. Proc Natl Acad Sci U S A.

[B39] Schmeck B, Zahlten J, Moog K, van Laak V, Huber S, Hocke AC, Opitz B, Hoffmann E, Kracht M, Zerrahn J, Hammerschmidt S, Rosseau S, Suttorp N, Hippenstiel S (2004). Streptococcus pneumoniae-induced p38 MAPK-dependent phosphorylation of RelA at the interleukin-8 promotor. J Biol Chem.

[B40] Guillot L, Le Goffic R, Bloch S, Escriou N, Akira S, Chignard M, Si-Tahar M (2005). Involvement of toll-like receptor 3 in the immune response of lung epithelial cells to double-stranded RNA and influenza A virus. J Biol Chem.

[B41] Zhang Z, Liu R, Noordhoek JA, Kauffman HF (2005). Interaction of airway epithelial cells (A549) with spores and mycelium of Aspergillus fumigatus. J Infect.

